# When are postpartum haemorrhages diagnosed? A nested observational study within the E-MOTIVE cluster-randomised trial

**DOI:** 10.1016/S2214-109X(25)00302-X

**Published:** 2025-10-15

**Authors:** Kristie-Marie Mammoliti, James Martin, Adam Devall, Christina Easter, Fernando Althabe, Adeosun Love Funmi, Rahmatu Yusuf, Fatima Abubakar, Lolade Christiana Arigbede, Jim Kelly Mugambi, Polycarp Oyoo, Masumbuko Sambusa, Akwinata Banda, Fawzia Samuels, Sara Willemse, Sibongile Doris Khambule, Hilal Mukhtar Shu'aib, Aminu Ado Wakili, Jenipher Okore, Ard Mwampashi, Mandisa Singata-Madliki, Edna Arends, Elani Muller, Hadiza Galadanci, Zahida Qureshi, Fadhlun Alwy Al-Beity, G Justus Hofmeyr, Sue Fawcus, Neil Moran, Alfred Osoti, George Gwako, Ioannis Gallos, Arri Coomarasamy

**Affiliations:** a College of Medicine and Health, University of Birmingham, Birmingham, UK; b UNDP–UNFPA–UNICEF–WHO–World Bank Special Programme of Research, Development, and Research Training in Human Reproduction, Department of Sexual and Reproductive Health and Research, World Health Organization, Geneva, Switzerland; c African Center of Excellence for Population Health and Policy, College of Health Sciences, Bayero University, Kano, Nigeria; d Department of Obstetrics and Gynecology, University of Nairobi, Nairobi, Kenya; e Department of Obstetrics and Gynecology, Muhimbili University of Health and Allied Sciences, Dar es Salaam, Tanzania; f Department of Obstetrics and Gynaecology, University of Cape Town, Cape Town, South Africa; g Effective Care Research Unit, University of the Witwatersrand, Johannesburg, South Africa; h Walter Sisulu University, Mthatha, South Africa; i Department of Obstetrics and Gynecology, University of Botswana, Gaborone, Botswana; j KwaZulu-Natal Department of Health, Pietermaritzburg, South Africa; k Department of Obstetrics and Gynaecology, Nelson Mandela School of Medicine, University of KwaZulu-Natal, Durban, South Africa; l Nuffield Department of Women's and Reproductive Health, John Radcliffe Hospital, University of Oxford, Oxford, UK

## Abstract

**Background:**

The definition of primary postpartum haemorrhage as blood loss of 500 mL or more within 24 h after vaginal birth underemphasises the early postpartum hours due to limited data on the timing of postpartum haemorrhage diagnosis. Understanding postpartum haemorrhage diagnosis timing and diagnostic methods is important for guiding diagnostic and therapeutic strategies. The E-MOTIVE trial evaluated early postpartum haemorrhage diagnosis and a bundled treatment approach, which resulted in a 60% relative reduction in adverse outcomes from bleeding compared with usual care. We aimed to compare timing from vaginal birth to postpartum haemorrhage diagnosis and the diagnostic methods used among four African countries implementing the intervention from the E-MOTIVE trial.

**Methods:**

Nested within the E-MOTIVE trial (NCT04341662), we conducted direct observations of health-care workers providing clinical care to postpartum women at 39 hospitals implementing the E-MOTIVE intervention across Nigeria, Kenya, Tanzania, and South Africa. One to two weeks of continuous observations were conducted from vaginal birth up to 2 h, between June 27, and Dec 18, 2022. Health-care workers were trained to use clinical judgement (ie, heavy vaginal blood loss, large blood clots expelled, or constant trickle) and various objective blood loss thresholds to diagnose postpartum haemorrhage. The objective blood loss thresholds used in Nigeria, Kenya, and Tanzania were 300 mL or more with at least one abnormal clinical sign (ie, pulse, blood pressure, uterine tone, and vaginal blood flow) or 500 mL or more. The objective blood loss threshold in South Africa was 500 mL or more. We descriptively analysed and compared timing from vaginal birth to postpartum haemorrhage diagnosis and diagnostic methods used between countries.

**Findings:**

Of 2578 women, 295 postpartum haemorrhages were diagnosed. The median time from vaginal birth to postpartum haemorrhage diagnosis was 15 min in Nigeria and Tanzania, 17 min in Kenya, and 30 min in South Africa. Diagnosis within 30 min ranged from 58% in South Africa to 86% in Tanzania. By 60 min, 96% to 100% of postpartum haemorrhages were diagnosed across all countries. All postpartum haemorrhages that required an intervention were diagnosed within 90 min. Nigeria, Kenya, and Tanzania commonly used blood loss of 300 mL or more combined with at least one abnormal clinical sign (47%, 65%, and 68%, respectively) while South Africa relied on a definition of 500 mL or more (81%) as the dominant diagnostic strategy.

**Interpretation:**

In countries where an objective blood loss threshold of 300 mL or more with at least one abnormal clinical sign was used, women received earlier interventions for postpartum bleeding, with a median time to diagnosis of 15–17 min. This was notably faster than in the country that predominantly used a 500 mL or more threshold, where the median time to diagnosis was 30 min. Regardless of whether the threshold was 300 mL or more with abnormal clinical signs or 500 mL or more alone, all postpartum haemorrhages were diagnosed within 90 min of vaginal birth.

**Funding:**

Bill & Melinda Gates Foundation and Ammalife.

## Introduction

Postpartum haemorrhage is the leading cause of maternal mortality worldwide, affecting approximately 14 million women, resulting in around 70 000 preventable deaths annually.^1^, ^2^, ^3^, ^4^, ^5^, ^6^ The burden of postpartum haemorrhage disproportionately impacts sub-Saharan Africa, where most postpartum haemorrhage-related maternal deaths occur.^7^ Currently, primary postpartum haemorrhage is defined as vaginal blood loss of 500 mL or more within 24 h of vaginal childbirth, despite limited supportive evidence.^7^, ^8^, ^9^

**Research in context**

**Evidence before this study**

Postpartum haemorrhage is defined by WHO as blood loss of 500 mL or more within the first 24 h after childbirth. Although most postpartum haemorrhage-related maternal deaths occur within 2–3 h of vaginal birth, there is limited evidence on how and when postpartum haemorrhage is diagnosed during this critical period. To explore the existing literature on the timing and methods of postpartum haemorrhage diagnosis, we conducted a PubMed search from database inception to Feb 1, 2022, using the terms ((“postpartum hemorrhage” OR “postpartum haemorrhage” OR “blood loss thresholds”) AND (“detection time”)). No publications were identified. A second and third search using the terms ((“postpartum hemorrhage” OR “postpartum haemorrhage” AND “blood loss thresholds”)), and ((“postpartum hemorrhage” OR “postpartum haemorrhage” OR “blood loss thresholds”) AND (“clinical signs”)) identified literature reviews, systematic reviews, and retrospective studies, but no prospective observational studies assessing diagnostic methods alongside diagnosis timing. Evidence remains limited on the interval between vaginal birth and postpartum haemorrhage diagnosis, particularly for blood loss thresholds below 500 mL and the integration of clinical signs into diagnostic approaches. During the baseline phase of the E-MOTIVE trial, the E-MOTIVE process evaluation involved stakeholder consultations and design workshops in each participating country in 2021 to assess the feasibility, acceptability, and local adaptation of proposed interventions. These informed pilot implementation strategies in three hospitals per country. Consensus was reached on postpartum haemorrhage diagnostic criteria: all countries agreed to use clinical judgement and a 500 mL or more threshold; in Nigeria, Kenya, and Tanzania, an additional threshold of 300 mL or more combined with at least one abnormal clinical sign or clinical concern was adopted. Addressing the gaps in evidence on early postpartum haemorrhage diagnosis timing and diagnostic methods—especially involving blood loss thresholds of 500 mL or less—is important for improving outcomes for women experiencing postpartum haemorrhage.

**Added value of this study**

The E-MOTIVE trial, which evaluated early postpartum haemorrhage diagnosis and a bundled treatment approach, showed a 60% relative reduction in severe postpartum haemorrhage, or laparotomy due to bleeding, or maternal death from bleeding compared with usual care. To the best of our knowledge, this study is the first to assess the time from vaginal birth to postpartum haemorrhage diagnosis through direct observations within a large, multi-country, cluster-randomised trial. Additionally, it examines the diagnostic methods and objective blood loss thresholds used for early postpartum haemorrhage diagnosis at the country level. A key contribution of our study is the analysis of country-level data, which showed variations in diagnosis times across countries. Health-care workers in Nigeria, Kenya, and Tanzania more frequently used the objective blood loss threshold of 300 mL or more combined with at least one abnormal clinical sign, reflecting the training they received. This approach led to timelier postpartum haemorrhage diagnosis, with a median time of 15–17 minutes compared with the 30 min using the 500 mL or more threshold. The 500 mL or more threshold and clinical judgment were used as secondary methods. In contrast, in South Africa, where the training focused on the 500 mL or more threshold and clinical judgment, the longest time to diagnosis was observed. All countries diagnosed postpartum haemorrhages within 90 min postpartum.

**Implications of all the available evidence**

The available evidence highlights the critical importance of timely postpartum haemorrhage diagnosis, prioritising the initial 90 min postpartum. Scaling up E-MOTIVE care should include a clear rationale for early postpartum haemorrhage diagnosis, incorporating objective blood loss thresholds of 300 mL or more combined with at least one abnormal clinical sign or clinical concern such as anaemia, as well as a 500 mL or more threshold as a safety net. Clinical judgment should complement these objective measures. Expanding the definition of postpartum haemorrhage to include lower, context-specific thresholds, with an emphasis on early diagnosis within the first 90 min postpartum, is important to improve maternal outcomes related to postpartum haemorrhage. Integrating these findings into clinical guidelines, clinical education (ie, obstetric and midwifery), and ongoing training for all relevant health-care professionals will help ensure timely postpartum haemorrhage diagnosis, contributing to improved maternal health outcomes, particularly in low-income and middle-income countries. Additionally, the use of calibrated blood loss measurement tools and globally aligned diagnostic criteria—while allowing for context-specific adaptations—can further enhance accuracy. These strategies are particularly important in low-income and middle-income countries, where improving early diagnosis and response can substantially impact maternal health.

Massive postpartum haemorrhage (ie, ≥1500 mL) usually occurs within the first hour postpartum,^10^ and most postpartum haemorrhage-related deaths follow within 2 to 3 hours.^11^, ^12^, ^13^ Data on postpartum haemorrhage-related timeframes exist but are limited, and the definition does not fully capture the critical importance of the early postpartum hours for mitigating postpartum haemorrhage-related maternal deaths. An early diagnosis, at a blood loss threshold of less than 500 mL might allow improved outcomes through earlier interventions.^14^

Pacagnella and colleagues^15^ conducted a systematic review to assess the relationship between blood loss and clinical signs, finding an association between increasing blood loss and changes in clinical parameters. However, the review recognised the challenge of establishing specific thresholds, as most studies do not have data on obstetric patients. More recent research highlights the potential value of the shock index, calculated by dividing heart rate by systolic blood pressure, in identifying women at risk of hypovolemic shock who require resuscitation.^16^ However, some authors caution against its use as a screening tool due to its low sensitivity.^16^, ^17^ Diagnosing postpartum haemorrhage only after a woman has already developed haemodynamic instability and hypovolemic shock might be too late to prevent worse outcomes such as morbidity and mortality.

Understanding the timing of blood loss in conjunction with other abnormal clinical signs is important for early diagnosis and timely intervention. Considering alternative blood loss thresholds with the assessment of the co-occurrence of abnormal clinical signs such as tachycardia and hypotension, atonic uterus, and increased vaginal blood flow could provide additional diagnostic value. Relying solely on an arbitrary blood loss volume of 500 mL or more, whether alone or in combination with haemodynamic instability, has been ineffective in reducing adverse postpartum haemorrhage outcomes.^1^ There is a need to explore and evaluate different diagnostic criteria and thresholds that incorporate alternative blood loss thresholds, timing, and abnormal clinical signs to improve the diagnosis of postpartum haemorrhage.

Understanding when and how postpartum haemorrhage is diagnosed is essential for health-care workers to allocate resources effectively and improve maternal outcomes. Despite the importance of early diagnosis,^18^ there is limited knowledge about the timing of postpartum haemorrhage diagnosis within the crucial initial hours postpartum. This gap highlights the need for research to inform clinical practice, improve early postpartum haemorrhage diagnosis, and enable prompt intervention.

We conducted a nested observational study within the E-MOTIVE trial (NCT04341662), a cluster-randomised intervention aimed at improving early postpartum haemorrhage diagnosis and management in women who had had a vaginal birth in low-income and middle-income countries (LMICs).^14^ The intervention focused on early postpartum haemorrhage diagnosis through a volumetric approach—objectively measuring blood loss in an obstetric drape with graduated measurement lines—and included a postpartum haemorrhage treatment bundle called MOTIVE (uterine massage, oxytocics, intravenous fluids, and examination of the genital tract) with an implementation strategy. The control group relied on subjective visual estimation of blood loss using a non-calibrated obstetric drape and management based on local guidelines. The trial showed a 60% reduction in severe postpartum haemorrhage (ie, ≥1000 mL), or laparotomy for bleeding, or maternal death from bleeding in the intervention group (E-MOTIVE care) compared with the control group (ie, usual care).

Our study aimed to address a crucial knowledge gap by evaluating the timing from vaginal birth to postpartum haemorrhage diagnosis and comparing timing and diagnostic methods between the four African countries involved in the E-MOTIVE intervention: Nigeria, Kenya, Tanzania, and South Africa. Through this, we hope to provide insights that guide health-care workers in optimising early postpartum haemorrhage diagnosis and inform guidelines to improve postpartum haemorrhage-related maternal outcomes in these regions.

## Methods

### Study design

We conducted a nested prospective observational study at 78 hospitals participating in the E-MOTIVE trial^14^ across Nigeria, Kenya, Tanzania, and South Africa from June 27, to Dec 18, 2022. This analysis focused specifically on observations from the 39 hospitals randomised into the intervention for the main trial of E-MOTIVE, to understand country-level differences in clinical practice across these four African countries.

The E-MOTIVE trial was approved by the relevant ethics and regulatory review committees of each country. Individual-level consent from women for observations was not obtained because they were not the target of the E-MOTIVE intervention, they were not interacted with for data collection, and no identifiable information was recorded. The study adhered to the principles of the Declaration of Helsinki, the Council for International Organizations of Medical Sciences International Ethical Guidelines, and the Ottawa Statement for the Ethical Design and Conduct of Cluster Randomised Trials.

### Postpartum haemorrhage diagnosis

During the 7-month implementation phase of the E-MOTIVE trial, a calibrated obstetric drape was introduced in 39 intervention hospitals. Marked with graduated lines in millilitres, it enabled objective cumulative blood loss measurement, facilitating early postpartum haemorrhage diagnosis. Health-care workers were also trained to conduct postpartum maternal assessments, including using the calibrated drape to support accurate postpartum haemorrhage diagnosis. Postpartum maternal assessments involved evaluating uterine tone, vaginal blood flow, and objective cumulative blood loss every 15 min for at least the first hour postpartum, as well as measuring vital signs (ie, blood pressure and pulse) at least once during the first hour and as needed during the second hour while the obstetric drape was applied.

During the E-MOTIVE trial baseline phase, 2-day stakeholder consultation and design workshops were held in Nigeria, Kenya, Tanzania, and South Africa.^19^ During these workshops, the feasibility, acceptability, and local adaptations of the implementation strategies were discussed and agreed upon through consensus with all stakeholders. In Nigeria, Kenya, and Tanzania, the consensus was to use a 300 mL or more blood loss threshold combined with at least one abnormal clinical sign or clinical concern, in addition to the 500 mL or more threshold (regardless of abnormal clinical signs) and clinical judgment. In contrast, South Africa maintained the use of the 500 mL or more threshold and clinical judgment. Following on from the consultation workshops, the diagnostic criteria applied during the implementation phase included clinical judgement, based on heavy vaginal blood loss, large blood clots expelled, constant trickle, and an objective blood loss threshold of 500 mL or more observed in the drape, regardless of clinical signs, or 300 mL or more in the drape combined with at least one abnormal clinical sign or additional clinical concern such as anaemia. The predefined abnormal clinical signs were a pulse of more than 100 beats per minute or an increase of 20 beats per minute or more from baseline; blood pressure if systolic was less than 100 mm Hg or decreased by 20 mm Hg from baseline; uterine tone if soft; vaginal blood flow if heavy flow vaginal blood loss or large blood clots expelled or a constant trickle. Details of the E-MOTIVE trial,^14^ and other analyses of this observational study^20^, ^21^ are published elsewhere.

### Nested study procedures

We observed health-care workers providing clinical care to women from vaginal birth until the obstetric drape was removed. To minimise selection bias and ensure comprehensive data collection, direct observations were conducted continuously throughout the day and night, including weekdays and weekends, over one to two weeks at each hospital. All women who had a vaginal birth at a hospital participating in the E-MOTIVE trial were eligible for inclusion in this study. The nested study did not impose any specific exclusion criteria; however, any woman excluded from the primary E-MOTIVE trial was automatically excluded from the nested study.

33 implementation and research midwives employed by the trial, all experienced and familiar with the E-MOTIVE intervention, were trained as observers. The implementation and research midwives, who acted as observers, occasionally transitioned to active clinical roles only if postpartum haemorrhage became life-threatening and required immediate intervention from all available clinically trained staff to ensure patient safety. A structured observation guide was used (appendix 1 p 2). Observations were documented on the paper-based guide and then transcribed into Research Electronic Data Capture. All data entries were reviewed daily (by K-MM) to ensure clinical coherence and address missing data.

The structured guide included patient characteristics, time of birth, time of drape funnel opening and removal, active management of the third stage, and postpartum maternal assessments, documented with start and stop timestamps. These assessments were documented on the blood loss monitoring chart specifically designed for the E-MOTIVE trial (appendix 1 p 2). For clinically diagnosed postpartum haemorrhages, data collected included the time of diagnosis, diagnostic methods and blood loss thresholds, treatments administered, and escalation of care procedures, all documented with start and stop timestamps. Medication doses and the health-care worker cadre responsible for each aspect of care were also recorded. Maternal age, gestational age at birth, singleton or multiple pregnancy, number of previous births at 24 weeks or older, previous caesarean section, postpartum haemorrhage in a previous pregnancy, episiotomy, perineal tear, balloon tamponade, laparotomy for bleeding, blood transfusion for bleeding, death due to bleeding, and final objective blood loss measure (source-verified) were obtained from the primary E-MOTIVE trial database.

### Outcomes

All observations in this analysis include only women with a diagnosed postpartum haemorrhage at hospitals implementing the E-MOTIVE intervention. Additionally, we analysed data from the E-MOTIVE trial to determine the number of postpartum haemorrhages diagnosed after the obstetric drape was removed (after observations were completed) until 24 h. The outcomes for the nested study were not pre-defined within the E-MOTIVE protocol. However, the outcomes drawn from the primary E-MOTIVE trial were pre-specified. The outcome measures reported in this analysis are: the time from vaginal birth to postpartum haemorrhage diagnosis, defined as a postpartum haemorrhage diagnosed by a health-care worker, regardless of final objective blood loss measure after removal of the drape during the observation period; the frequency and timing of diagnostic methods used including clinical judgement, objective blood loss according to the calibrated drape of 500 mL or more, or 300 mL or more combined with at least one abnormal clinical sign during the observation period; and the frequency of postpartum haemorrhage diagnosed after the observation period, from removal of the obstetric drape until 24 h postpartum, at which point at least one of the following interventions (due to bleeding) were recorded in the E-MOTIVE trial: laparotomy, balloon tamponade, blood transfusion, or death.

### Statistical analysis

A subgroup representing around 2·5% of vaginal births (of 99 659 women) was observed in the intervention group during E-MOTIVE implementation, within the trial's predefined timeframe. Descriptive analysis was performed, overall and stratified by country (Nigeria, Kenya, Tanzania, and South Africa). Continuous measures were reported as medians and IQR due to skewness, and categorical data were reported as frequencies and percentages. All analyses used complete case analysis with minimal missing data. Differences in time from vaginal birth to postpartum haemorrhage diagnosis between countries were assessed using a Kruskal-Wallis test, due to the skewness of the data. STATA 18 was used to conduct this analysis.

### Role of the funding source

The funders of the study had no role in study design, data collection, data analysis, data interpretation, or writing of the report.

## Results

**Figure 1:**
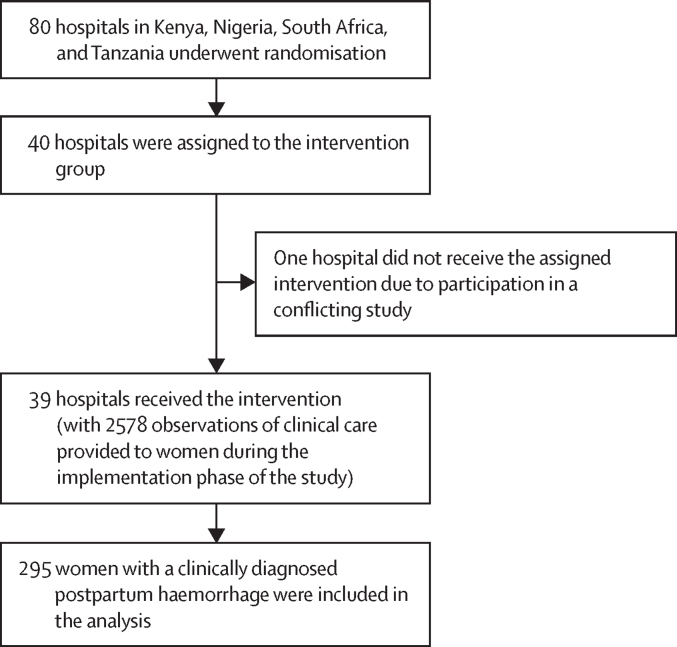
CONSORT diagram

During the E-MOTIVE trial, 80 hospitals across Nigeria, Kenya, Tanzania, and South Africa were randomised, with 40 assigned to the E-MOTIVE care intervention and 40 to usual care. One hospital in the intervention group was excluded due to the introduction of a conflicting programme (the same was the case for the usual care group). Our analysis, therefore, focused on the 39 hospitals that implemented the E-MOTIVE care intervention. At these 39 hospitals, we conducted 2578 direct observations (ie, 2578 women) of health-care workers providing clinical care to women from vaginal birth until the removal of the obstetric drape. Among these observations, 295 (11·4%) women experiencing postpartum haemorrhage were clinically diagnosed by health-care workers (figure 1).

The baseline characteristics of the observed women were generally similar across countries, with a few notable differences (table 1, appendix 1 pp 2–4). In South Africa, seven (12%) of 57 women had a previous caesarean section and 11 (19%) were diagnosed with HIV, compared with less than 5% for both in other countries. In Nigeria, 14 (10%) of 141 women had a history of postpartum haemorrhage, compared with less than 2% elsewhere. Haemoglobin testing was performed in more than 73% of women in Kenya, Tanzania, and South Africa, but only 16 (11%) of 141 women in Nigeria. When tested, women in Nigeria had the lowest average haemoglobin at 94 g/L, compared with between 111 g/L and 113 g/L in the other countries. Labour augmentation occurred in 15 (11%) of 141 women in Nigeria and in five (7%) of 75 women in Kenya, while it was less common in Tanzania and South Africa, occurring in fewer than 2% of the women. Vaginal or perineal tears varied across countries, with the highest occurrence in South Africa (26 [46%] of 57), closely followed by Kenya (33 [44%] of 75), Tanzania (eight [36%] of 22), and Nigeria (32 [23%] of 141). Misoprostol for active management of the third stage of labour was administered in 116 (82%) of 141 women in Nigeria but to fewer than 5% in the other countries. Manual removal of the placenta occurred in seven (9%) of 75 of women in Kenya, three (5%) of 57 in South Africa, and less than 2% in Tanzania and Nigeria. The placenta was examined for completeness in more than 97% of the women in Kenya and South Africa, compared with 16 (73%) women in Tanzania and 32 (23%) in Nigeria.

**Table 1:** Baseline characteristics

	**Kenya (n=75)**	**Nigeria (n=141)**	**South Africa (n=57)**	**Tanzania (n=22)**	**Overall (N=295)**
**Pregnancy information**					
Maternal age, years	25 (21–28)	26 (22–30)	26 (22–31)	25 (18–28)	25 (21–30)
Gestational age at birth, months	38 (37–40)	38 (36–39)	39 (38–40)	39 (38–40)	38 (37–40)
Previous caesarean section	2 (3%)	2 (1%)	7 (12%)	1 (5%)	12 (4%)
**Health conditions**					
HIV	1 (1%)	0	11 (19%)	0	12 (4%)
Hypertension	4 (5%)	7 (5%)	1 (2%)	0	12 (4%)
Malaria	0	2 (1%)	0	1 (5%)	3 (1%)
Diabetes	0	1 (1%)	0	0	1 (0%)
**Pregnancy, labour, birth risk factors**					
Previous postpartum haemorrhage	1 (1%)	14 (10%)	1 (2%)	0	16 (5%)
Frequency of haemoglobin testing in pregnancy	64 (85%)	16 (11%)	55 (96%)	16 (73%)	151 (51%)
Haemoglobin levels, g/L	111 (105–120)	94 (107–113)	113 (100–124)	112 (99–121)	112 (102–120)
Placental abruption	0	7 (5%)	0	0	7 (2%)
Pregnancy induced hypertension	3 (4%)	14 (10%)	5 (9%)	0	22 (7%)
Pre-eclampsia	1 (1%)	11 (8%)	3 (5%)	1 (5%)	16 (5%)
Eclampsia	0	3 (2%)	0	1 (5%)	4 (1%)
Antepartum haemorrhage	0	5 (4%)	0	0	5 (2%)
Induction of labour	2 (3%)	8 (6%)	11 (19%)	0	21 (7%)
Augmentation of labour	5 (7%)	15 (11%)	1 (2%)	0	21 (7%)
Episiotomy	14 (19%)	32 (23%)	14 (25%)	0	60 (20%)
Vaginal or perineal tear	33 (44%)	32 (23%)	26 (46%)	8 (36%)	99 (34%)
**Active management of third stage of labour: medicines administered**					
Oxytocin	75 (100%)	137 (97%)	57 (100%)	22 (100%)	291 (99%)
Misoprostol	1 (1%)	116 (82%)	0	1 (5%)	118 (40%)
**Active management of third stage of labour: management of the placenta**					
Controlled cord traction performed	68 (91%)	138 (98%)	54 (95%)	22 (100%)	282 (96%)
Manual removal of placenta performed	7 (9%)	3 (2%)	3 (5%)	0	13 (4%)
Placenta checked for completeness	73 (97%)	32 (23%)	56 (98%)	16 (73%)	177 (60%)

Data are n (%) or median (IQR).

**Figure 2:**
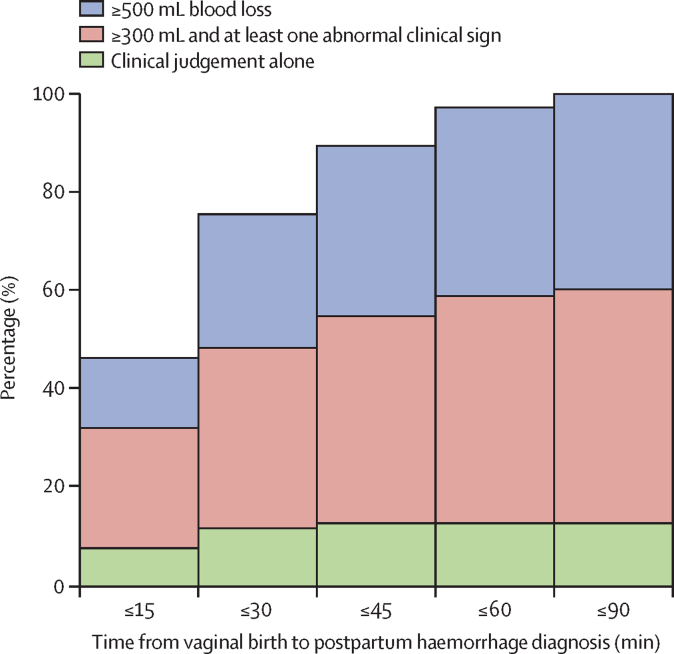
Elapsed time from vaginal birth to postpartum haemorrhage diagnosis by diagnostic methods and blood loss thresholds overall

Overall, the median time from vaginal birth to postpartum haemorrhage diagnosis was 17 min (IQR 11–30; table 2). Elapsed time from vaginal birth to postpartum haemorrhage diagnosis was categorised into 0–30, 0–60, and 0–90 minutes based on clinical relevance for early postpartum haemorrhage diagnosis. The diagnosis rates were as follows: 223 (76%) of 295 of women with postpartum haemorrhage were diagnosed within 30 min; 287 (97%) within 60 min; and 295 (100%) within 90 min (table 2). The diagnostic methods varied: the most frequently used was a blood loss threshold of 300 mL or more combined with at least one abnormal clinical sign (140 [47%] of 295), followed by a blood loss threshold of 500 mL or more (117 [40%]). Clinical judgement was used least often (38 [13%]; table 2; figure 2).

**Table 2:** Birth to postpartum haemorrhage detection by diagnostic methods and blood loss thresholds

		**Kenya (n=75)**	**Nigeria (n=141)**	**South Africa (n=57)**	**Tanzania (n=22)**	**Overall (N=295)**
Elapsed time from birth to postpartum haemorrhage detection, min						
	0–30	60 (80%)	111 (79%)	33 (58%)	19 (86%)	223 (76%)
	0–60	74 (99%)	136 (96%)	55 (96%)	22 (100%)	287 (97%)
	0–90	75 (100%)	141 (100%)	57 (100%)	22 (100%)	295 (100%)
Detected postpartum haemorrhages, number of women		75/295 (25%)	141/295 (48%)	57/295 (19%)	22/295 (8%)	295 (100%)
Detected postpartum haemorrhages, time from vaginal birth in min		17 (11–30)	15 (10–30)	30 (18–35)*	15 (9–30)	17 (11–30)
Clinical judgement, number of women		12 (16%)	20 (14%)	1 (2%)	5 (23%)	38 (13%)
Clinical judgement, time from vaginal birth in min		17 (13–21)	8 (5·5–13·5)	31 (31–31)	30 (9–30)	12 (7–20)
≥300 mL†, number of women		49 (65%)	66 (47%)	10 (18%)	15 (68%)	140 (47%)
≥300 mL†, time from vaginal birth in min		17 (12–30)	15 (9–30)	31 (15–39)	15 (8–30)	15 (10–30)
≥500 mL, number of women		14 (19%)	55 (39%)	46 (81%)	2 (9%)	117 (40%)
≥500 mL, time from vaginal birth in min		21 (17–42)	17 (15–35)	30 (18–35)	30 (15–45)	21 (15–35)

Data are n (%), n/N (%), or median (IQR).

* p≤0·0003 in comparison to the median time to detection in the other countries.

† ≥300 mL combined with at least one abnormal clinical sign.

**Figure 3:**
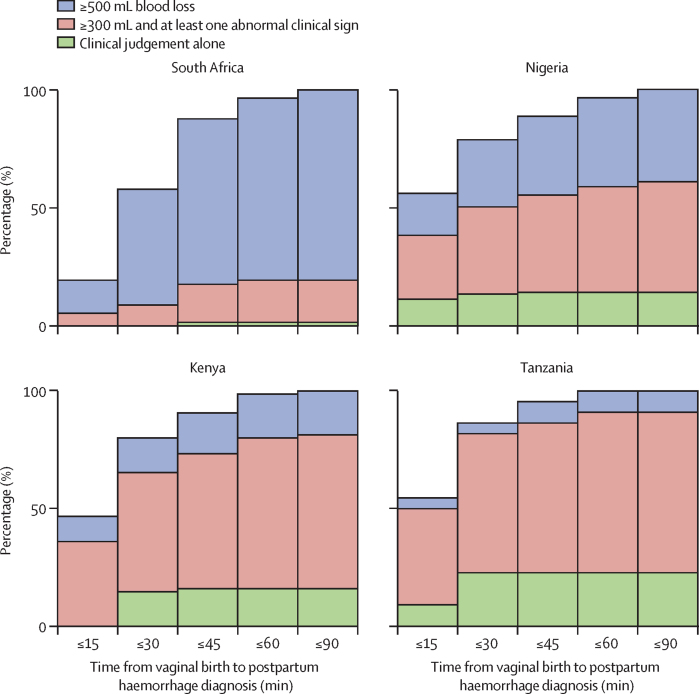
Elapsed time from vaginal birth to postpartum haemorrhage diagnosis by diagnostic methods and blood loss thresholds by country

Tanzania had the fastest diagnosis time from vaginal birth, with a median time of 15 min (IQR 9–30), 19 (86%) of 22 diagnosed within 30 min, and all women experiencing postpartum haemorrhage diagnosed by 60 min (table 2; figure 3); followed by Nigeria who had a median diagnosis time of 15 min (10–30), with 111 (79%) of 141 postpartum haemorrhages diagnosed within 30 min, 136 (96%) of 141 by 60 min, and all by 90 min. Kenya showed a similar pattern with a median time of 17 min (11–30), 60 (80%) of 75 haemorrhages diagnosed within 30 min, 74 (99%) by 60 min, and all by 90 min. South Africa had the longest median diagnosis time of 30 min (18–35), with the lowest proportion diagnosed within 30 min (33 [58%] of 57), though 55 (96%) haemorrhages were diagnosed within 60 min, and all were within 90 min after vaginal birth. There was strong statistical evidence of a difference in the time from birth to postpartum haemorrhage diagnosis across countries (p value ≤0·0003), indicating that at least one country's median time to diagnosis differed from the others (table 2).

The diagnostic methods and blood loss thresholds used to diagnose postpartum haemorrhage varied across countries. South Africa predominantly used the 500 mL or more blood loss threshold (46 [81%] of 57), whereas Nigeria, Kenya, and Tanzania, more frequently used the 300 mL or more blood loss threshold combined with at least one abnormal clinical sign (66 [47%] of 141, 49 [65%] of 75, and 15 [68%] of 22, respectively). Clinical judgement alone was used in a smaller proportion of women experiencing postpartum haemorrhage, ranging from one (2%) of 57 in South Africa to five (23%) of 22 in Tanzania (table 2; figure 3). There was no postpartum haemorrhage that required an intervention diagnosis between the removal of the obstetric drape to 24 h postpartum in any of the countries.

## Discussion

Our findings show that women experiencing postpartum haemorrhage had an average diagnosis time of 17 min after vaginal birth, with none diagnosed beyond 90 min postpartum. However, significant variation in diagnosis times were observed between countries, with Tanzania being the most efficient (15 min) and South Africa having the longest diagnosis time (30 min). Despite these differences, most women experiencing postpartum haemorrhage were diagnosed within 60 min, and all within 90 min after birth during the observation period, showing the effectiveness of the early diagnosis approach implemented at E-MOTIVE care hospitals. The significant variation in diagnosis times (p value ≤0·0003) suggests the presence of country-specific factors influencing diagnosis speed.

The variation in diagnostic methods and blood loss thresholds used across countries likely contributed to differences in diagnosis times. South Africa predominantly relied on a 500 mL or more threshold, whereas Nigeria, Kenya, and Tanzania more often applied a 300 mL or more threshold combined with at least one abnormal clinical sign or additional clinical concern such as anaemia. These findings suggest integrating a lower blood loss threshold with at least one abnormal clinical sign or clinical concern such as anaemia, could enhance early postpartum haemorrhage diagnosis and facilitate timely management, optimising intervention within 30 min postpartum while ensuring diagnosis remains within 90 min.

In some situations, treatment for postpartum haemorrhage can be initiated based on clinical judgement alone, without an immediate assessment of blood loss. For example, if a woman has severe vaginal bleeding, is shocked (ie, tachycardic and hypotensive), or becomes unconscious, a postpartum haemorrhage diagnosis can be made using clinical judgement alone. A diagnosis of postpartum haemorrhage can also be made using clinical judgment in less extreme situations, for example, when a woman with risk factors such as anaemia has moderate but persistent bleeding that causes concern to the health-care worker. Therefore, postpartum haemorrhage diagnosis using clinical judgement was used as per usual clinical practice.

Relying solely on objective criteria might lead to missed postpartum haemorrhages, delaying necessary interventions. Integrating clinical judgment alongside standardised thresholds ensures a more comprehensive and responsive approach to early diagnosis and management, ultimately improving maternal outcomes.

Adopting a lower threshold for postpartum haemorrhage diagnosis enabled earlier MOTIVE bundle use. The E-MOTIVE process evaluation found that early detection and the MOTIVE bundle together reduced workload through a structured approach, boosting confidence and decreasing stress for staff.^20^ Although, some midwives initially found added responsibility challenging, over time they adapted and felt more empowered. These opinions were collected through interviews and surveys. Notably, earlier diagnosis and management might reduce progression to severe postpartum haemorrhage, potentially decreasing the need for intensive interventions and resource use. Managing postpartum haemorrhage before a woman becomes haemodynamically unstable and requires urgent, life-saving interventions could further reduce the overall burden on health-care providers.

Since no postpartum haemorrhages required an intervention between the obstetric drape removal and 24 h postpartum, prioritising the first 90 min postpartum as the critical window for early postpartum haemorrhage diagnosis is essential to reducing postpartum haemorrhage-related adverse outcomes for women. To the best of our knowledge, no direct observational studies have evaluated the timing from vaginal birth to postpartum haemorrhage diagnosis, and data on blood loss thresholds less than 500 mL were scarce.^18^ The WOMAN-2 trial, a randomised, double-blind, placebo-controlled study investigated postpartum haemorrhage prophylaxis using tranexamic acid in women who were anaemic, provided some insights into diagnosis timing. It reported a median time of 18·5 min to postpartum haemorrhage diagnosis in women with moderate anaemia and 13 min in those with severe anaemia,^22^ aligning with our findings.

A key strength of this study is its integration within the multi-country E-MOTIVE cluster-randomised trial across diverse health-care settings. The use of implementation and research midwives, already familiar with the hospital environments, minimised potential disruptions in providing health care and mitigated the Hawthorne effect.^23^ Additionally, previous research suggests that health-care workers become less conscious of being observed over time, resulting in more natural behaviour.^24^, ^25^ Although observer bias could not be fully eliminated, trial-employed staff conducted the observations to reduce this risk by ensuring consistency and standardisation.

One notable limitation of this study, which could not be mitigated, is the relatively low number of observations by country and differences in baseline characteristics between countries. Additionally, although no woman required postpartum haemorrhage treatment after 2 h, some women might have had moderate bleeding that met postpartum haemorrhage criteria if blood loss had been assessed beyond that time. Furthermore, this study only assessed the E-MOTIVE intervention in African settings; its generalisability to other LMICs and high-income contexts warrants evaluation. Finally, in South Africa, postpartum maternal assessments and postpartum haemorrhage diagnosis were done by overstretched routine staff, as most research staff were non-clinicians. The E-MOTIVE process evaluation found that routine staff primarily diagnosed postpartum haemorrhage in South Africa and Kenya, whereas in Nigeria and Tanzania, research staff did so about half the time.^20^ Addressing clinical staffing shortages is essential for timely postpartum haemorrhage diagnosis, especially in high-burden settings; this study offers key insights into its timing and diagnostic methods across diverse settings.

The criteria for defining postpartum haemorrhage are often standardised to aid clinical decision making and service delivery. However, the volume of blood loss that can be tolerated varies depending on a woman's individual health status; for example, a healthy woman might tolerate moderate loss, whereas a severely anaemic woman might experience life-threatening consequences from the same amount. Thus, considering the contextual factors in the diagnosis and management of individual women is important. Our proposed threshold of 300 mL or more blood loss with at least one abnormal clinical sign is intended to aid earlier recognition of clinically important bleeding, particularly in settings in which delayed response can lead to adverse outcomes. This approach prioritises early postpartum maternal assessments and response to physiological signs of compromise. Further work is needed to determine whether this threshold could inform a revised definition of postpartum haemorrhage and to evaluate its diagnostic performance and clinical use in diverse settings.

Further research should examine causes of these variations and refine clinical protocols, particularly in LMICs in which timely response is essential. WHO is undertaking a rigorous reappraisal of the definition of postpartum haemorrhage. Our study provides valuable insights into postpartum haemorrhage diagnosis timing, diagnostic methods, and blood loss thresholds at the country level, which contributed to a significant improvement in the primary outcomes of the E-MOTIVE trial, detailed earlier in this Article.^14^ These findings support ongoing efforts to optimise early postpartum haemorrhage diagnosis and improve maternal outcomes in high burden settings.

### Contributors

### Equitable partnership declaration

### Data sharing

The study protocol, study instruments, and de-identified observation data, and the statistical code underlying the results reported in this Article will be made available after de-identification, upon request to the corresponding author immediately following publication until 5 years following publication. A data sharing agreement will require a commitment to using the data only for specified research purposes, to researchers who provide a methodologically sound proposal, to securing the data appropriately, and to destroying the data after a nominated period.

## Declaration of interests

GJH reports conceiving a re-usable device for postpartum blood loss monitoring, the Maternawell Tray, which is marketed by Maternova, a global women's health solutions company who hold the intellectual property. GJH benefited from consulting fees in the past as an inventor but receives no current income nor prospect of future income from this project. All other authors declare no competing interests.

## Supplementary Materials

Supplementary appendix 1

Equitable Partnership Declaration
