# Supplementary material for: When are postpartum haemorrhages diagnosed? A nested observational study within the E-MOTIVE cluster-randomised trial
Source: Lancet Glob Health. 2025 Oct 15;13(11):e1946–54. doi: 10.1016/S2214-109X(25)00302-X (PMC12535819; doi:10.1016/S2214-109X(25)00302-X)
Supplement: Supplementary appendix 1 [file mmc1.pdf]

THE LANCET
Global Health

Supplementary appendix 1

This appendix formed part of the original submission and has been peer reviewed. We post it as supplied by the authors.

Supplement to: Mammoliti K-M, Martin J, Devall A, et al. When are postpartum haemorrhages diagnosed? A nested observational study within the E-MOTIVE cluster-randomised trial. *Lancet Glob Health* 2025; **13:** e1946–54.

Table of Contents

1: Documentation .... Page 2

2: All baseline characteristics table .... Page 2

3: E-MOTIVE trial .... Page 5

1: Documentation

Observation guide
E-MOTIVE care - https://osf.io/7xmgc

Blood loss monitoring chart
Nigeria, Kenya, and Tanzania - https://osf.io/j4uas
South Africa - https://osf.io/sy4h7

2: All baseline characteristics table

Table 1: All baseline characteristics

..	Kenya	Nigeria	South Africa	Tanzania	Total
..	N=75	N=141	N=57	N=22	N=295
**Pregnancy information**	..	..	..	..	..
Maternal age	..	..	..	..	..
mean (SD)	25 (5·4)	26.65 (6·2)	26·4 (6·3)	26·1 (8·1)	26·1 (6·2)
median[IQR]	25 [21-28]	26 [22-30]	26 [22-31]	24·5 [18-28]	25 [21-30]
Gestational age at birth	..	..	..	..	..
mean (SD)	38·5 (2.4)	37·6 (2·8)	38·9 (2·2)	39·1 (1·4)	38·2 (2·6)
median[IQR]	38 [37-40]	38 [36-39]	39 [38-40]	39 [38-40]	38 [37-40]
UNKNOWN gestational age, (n, [%])	4 (5·3)	14 (9·9)	2 (3·5)	2 (9·1)	22 (7·5)
Type of pregnancy	..	..	..	..	..
Singleton, (n, [%])	70 (93·3)	129 (91·5)	57 (100)	20 (90·9)	276 (93·6)
Twin, (n, [%])	5 (67)	12 (8·5)	0 (0)	2 (9·1)	19 (6.4)
Number of previous births ≥ 24 weeks gestation, (n, [%])	75 (100)	136 (96·5)	57 (100)	22 (100)	290 (98·3)
Unknown number of previous births ≥ 24 weeks, (n, [%])	0 (0)	5 (3·5)	0 (0)	0 (0)	5 (1·7)
Previous caesarean section, (n, [%])	2 (2·7)	2 (1·4)	7 (12·3)	1 (4·5)	12 (4·1)
Previous postpartum haemorrhage, (n, [%])	1 (1·3)	14 (9·9)	1 (1·8)	0 (0)	16 (5·4)
**Health conditions**	..	..	..	..	..
Body Mass Index	..	..	..	..	..
mean (SD)	26·1 (4·4)	27·2 (6·1)	28·9 (6·1)	23·5 (1·4)	27·3 (5·6)
median[IQR]	25·1 [23·3-28·3]	26·9 [21.6-31·5]	28·3 [23·7-34·5]	23·3 [22·4-24·1]	26·4 [22·9-31·3]
< 18.5 (n, [%])	2 (2·7)	1 (0·7)	1 (1·8)	0 (0)	4 (1·4)

18.5 – 24.9 (n, [%])	12 (16)	14 (9·3)	15 (26·3)	11 (50)	52 (17·6)
≥ 25 (n, [%])	23 (30·7)	26 (18·4)	40 (70·2)	1 (4.5)	90 (30·5)
Hypertension, frequency, (n, [%])	4 (5·3)	7 (5)	1 (1·8)	0 (0)	12 (4·2)
Diabetes, frequency, (n, [%])	0 (0)	1 (0·7)	0 (0)	0 (0)	1 (0·3)
Autoimmune disease, (n, [%])	0 (0)	0 (0)	1 (1·8)	0 (0)	1 (0·3)
Sexually transmitted infection, (n, [%])	1 (1·3)	1 (0·71)	3 (5·3)	0 (0)	5 (1·7)
Kidney disease, (n, [%])	0 (0)	1 (0·7)	0 (0)	0 (0)	1 (0·3)
Human Immunodeficiency Virus, (n, [%])	1 (1·3)	0 (0)	11 (19·3)	0 (0)	12 (4·1)
Malaria, (n, [%])	0 (0)	2 (1·4)	0 (0)	1 (4·5)	3 (1)
Unbooked to attended hospital, (n, [%])	0 (0)	14 (9·9)	0 (0)	0 (0)	14 (4·7)
Pyelonephritis, (n, [%])	0 (0)	1 (0·7)	0 (0)	0 (0)	1 (0·3)
Pregnancy, labour, birth risk factors	··	··	··	····	··
Haemoglobin tested in pregnancy, (n, [%])	64 (85·3)	16 (11·4)	55 (96·5)	16 (72·7)	151 (51·2)
Haemoglobin	··	··	··	··	··
mean (SD)	109·5 (18·4)	103·3 (13·7)	113·4 (15·6)	108 (14·8)	110·1 (16·7)
median[IQR]	111 [105-120]	94 [107-113]	113 [100-124]	112 [98·5-121]	112 [102-120]
Taking iron tablets for > 1 month in pregnancy, (n, [%])	66 (88)	43 (30·5)	44 (77·2)	7 (31·8)	171 (58)
Previous neonatal death, (n, [%])	1 (1·3)	11 (7·8)	1 (1·8)	0 (0)	13 (4·4)
Hookworm treated in pregnancy, (n, [%])	19 (25·3)	0 (0)	0 (0)	15 (68·2)	34 (11·5)
Placenta previa or low lying, accreta, increta or percreta, (n, [%])	1 (1·3)	0 (0)	0 (0)	0 (0)	1 (0·3)
Polyhydramnios, (n, [%])	0 (0)	1 (0·7)	0 (0)	0 (0)	1 (0·3)
Placental abruption, (n, [%])	0 (0)	7 (5)	0 (0)	0 (0)	7 (2·4)
Chorioamnionitis, (n, [%])	0 (0)	0 (0)	1 (1·8)	0 (0)	1 (0·3)
Pregnancy induced hypertension, (n, [%])	3 (4)	14 (9·9)	5 (8·8)	0 (0)	22 (7·5)
Pre-eclampsia, (n, [%])	1 (1·3)	11 (7·8)	3 (5·3)	1 (4·5)	16 (5·4)
Eclampsia, (n, [%])	0 (0)	3 (2·1)	0 (0)	1 (4·5)	4 (1·4)
Antepartum haemorrhage, (n, [%])	0 (0)	5 (3·5)	0 (0)	0 (0)	5 (1·7)
Febrile in labour (temperature >38°C), (n, [%])	0 (0)	1 (0·7)	0 (0)	1 (4·5)	2 (0·7)
Pushing > 60 minutes, (n, [%])	1 (1·3)	3 (2·1)	4 (7)	0 (0)	8 (2·7)
Induction of labour, (n, [%])	2 (2·7)	8 (5·7)	11 (19·3)	0 (0)	21 (7·1)
Augmentation of labour, (n, [%])	5 (6·7)	15 (10·6)	1 (1·8)	0 (0)	21 (7·1)

Received antibiotics in labour, (n, [%])	0 (0)	3 (2·1)	6 (10·5)	0 (0)	9 (3·1)
Meconium liquor, (n, [%])	2 (27)	0 (0)	10 (17·5)	0 (0)	12 (4·1)
Malpresentation or malposition, (n, [%])	0 (0)	0 (0)	1 (1·8)	0 (0)	1 (0·3)
Episiotomy, (n, [%])	14 (18·7)	32 (22·7)	14 (24·6)	0 (0)	60 (20·3)
Vaginal/Perineal tear, (n, [%])	33 (44)	32 (22·7)	26 (45·6)	8 (36·4)	99 (33·6)
Shoulder dystocia, (n, [%])	0 (0)	1 (0·7)	3 (5·3)	0 (0)	4 (1·4)
Intrauterine fetal death, (n, [%])	0 (0)	2 (1·4)	0 (0)	0 (0)	2 (0·7)
obstructed labour, (n, [%])	0 (0)	1 (0·7)	0 (0)	0 (0)	1 (0·3)
Baby 1 - mode of birth	··	··	··	··	··
Spontaneous vaginal, (n, [%])	75 (100)	140 (99·3)	56 (98·2)	22 (100)	293 (99·3)
Ventouse, (n, [%])	0 (0)	1 (0·7)	1 (1·8)	0 (0)	2 (0·7)
Baby 2 - mode of birth	··	··	··	··	··
Spontaneous vaginal, (n, [%])	5 (6·7)	12 (8·5)	0 (0)	2 (9·1)	19 (6·4)
Study procedure	··	··	··	··	··
Time from vaginal birth to drape funnel opening (minutes)	··	··	··	··	··
mean (SD)	2 (1)	1 (0·7)	1·8 (3)	1·2 (3)	1·9 (1·5)
median[IQR]	2 [1-3]	1 [1-1]	2 [5-0]	2 [4-0]	1 [1-3]
**AMTSL: Medicines administered**	··	··	··	··	··
Oxytocin, (n/N, %)	75 (100)	137 (97·2)	57 (100)	22 (100)	291 (98·7)
Misoprostol, (n/N, %)	1 (1·3)	116 (82·3)	0 (0)	1 (4·5)	118 (40)
Ergometrine, (n/N, %)	0 (0)	1 (0·7)	0 (0)	0 (0)	1 (0.3)
Carbetocin, (n/N, %)	0 (0)	4 (2·8)	0 (0)	0 (0)	4 (1·4)
**AMTSL: Management of the placenta**	··	··	··	··	··
Controlled cord traction performed, (n/N, %)	68 (90·7)	138 (97·9)	54 (94·7)	22 (100)	282 (95·6)
Manual removal of placenta performed, (n/N, %)	7 (9·3)	3 (2·1)	3 (5·3)	0 (0)	13 (4·4)
Placenta checked, (n/N, %)	73 (97·3)	32 (22·7)	56 (98·2)	16 (72·7)	177 (60)

CAPTION: SD = standard deviation; IQR = interquartile range; n= number; N=total number; % = percentage; AMTSL= Active management of third stage of labour

3: E-MOTIVE trial

Design: Multi-country, parallel cluster randomised trial with a baseline control phase, along with mixed-methods and health economic evaluation

Population: All women giving vaginal birth at the study facilities

Intervention: Early detection of PPH and treatment bundle consisting of uterine Massage, Oxytocic drugs, Tranexamic acid, IV fluids and Examination & Escalation

Comparator: Usual care with dissemination of the current guidelines (treatment may include some or all of the components of the treatment bundle)

Outcome: Primary outcome: Composite of severe PPH (blood loss ≥1000 ml) or postpartum laparotomy for bleeding or postpartum maternal death from bleeding

Key findings

A primary-outcome event (a composite of primary severe postpartum haemorrhage (PPH) defined as blood loss ≥1000 ml following a vaginal birth, postpartum laparotomy for bleeding or postpartum maternal death from bleeding) occurred in 794 of 48,678 patients (1.6%) in the intervention group and in 2139 of 50,044 (4.3%) in the usual-care group (risk ratio, 0.40; 95% confidence interval [CI], 0.32 to 0.50; P<0.001). This represents a 60% relative reduction in the risk of the primary outcome. PPH was detected in 93.1% of the patients in the intervention group and in 51.1% of those in the usual-care group (rate ratio, 1.58; 95% CI, 1.41 to 1.76), and adherence to the treatment bundle was 91.2% and 19.4%, respectively (rate ratio, 4.94; 95% CI, 3.88 to 6.28). The risk of the primary outcome in the intervention group progressively decreased with time after randomization, from a mean of 3.8% during the baseline pre-randomization phase to 1.1% by the last month of the implementation phase (Figure S1).

Figure S1. Patients with primary outcome event during the baseline, transition, and implementation phases of the E-MOTIVE trial.

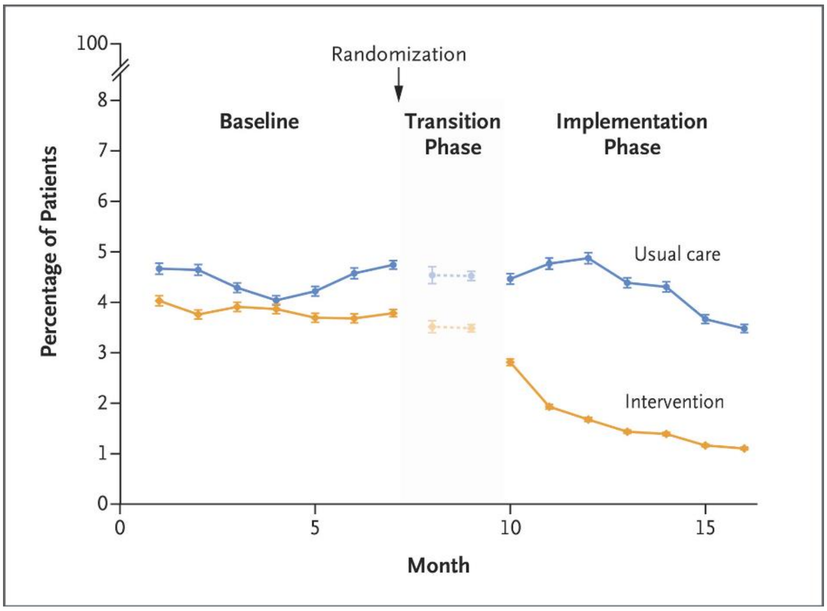

The median blood loss was 160 ml (interquartile range, 100 to 280) in the intervention group and 220 ml (interquartile range, 120 to 380) in the usual-care group. PPH (defined as blood loss of ≥500 ml) was

diagnosed in 8.5% of the patients in the intervention group and in 16.7% of those in the usual-care group (risk ratio, 0.51; 95% CI, 0.44 to 0.60), and severe PPH (defined as blood loss of ≥1000 ml) in 1.6% and 4.3%, respectively (risk ratio, 0.39; 95% CI, 0.31 to 0.49). Postpartum blood transfusion for bleeding was used in 1.2% of the patients in the intervention group and in 1.9% of those in the usual-care group (risk ratio, 0.71; 95% CI, 0.55 to 0.90). There were 17 maternal deaths in the intervention group and 28 deaths in the usual-care group (risk ratio, 0.73; 95% CI, 0.40 to 1.31). A total of 12 and 18 of these deaths, respectively, were attributed to postpartum bleeding. There were few cases of laparotomy, compression sutures, uterine-artery ligation or hysterectomy, a situation that limited meaningful comparisons between the trial groups.

Reference

Gallos I, Devall A, Martin J, Middleton L, Beeson L, et al. Randomized Trial of Early Detection and Treatment of Postpartum Hemorrhage. N Engl J Med. 2023 Jul 6;389(1):11-21.

Available at: https://www.nejm.org/doi/full/10.1056/NEJMoa2303966

Supplementary materials:
https://www.nejm.org/doi/suppl/10.1056/NEJMoa2303966/suppl_file/nejmoa2303966_appendix.pdf
