# Supplementary material for: When are postpartum haemorrhages diagnosed? A nested observational study within the E-MOTIVE cluster-randomised trial
Source: Lancet Glob Health. 2025 Oct 15;13(11):e1946–54. doi: 10.1016/S2214-109X(25)00302-X (PMC12535819; doi:10.1016/S2214-109X(25)00302-X)
Supplement: Equitable Partnership Declaration [file mmc2.pdf]

THE LANCET
Global Health

Supplementary appendix 2

This Equitable Partnership Declaration (EPD) was submitted by the authors, and we reproduce it as supplied. It has not been peer reviewed. *The Lancet*'s editorial processes have not been applied to the EPD.

Supplement to: Mammoliti K-M, Martin J, Devall A, et al. When are postpartum haemorrhages diagnosed? A nested observational study within the E-MOTIVE cluster-randomised trial. *Lancet Glob Health* 2025; **13:** e1946–54.

Equitable Partnership Declaration questions

Researcher considerations

1. Please detail the involvement that researchers who are based in the region(s) of study had during a) study design; b) clinical study processes, such as processing blood samples, prescribing medication, or patient recruitment; c) data interpretation; and d) manuscript preparation, commenting on all aspects. If they were not involved in any of these aspects, please explain why.

   This question is intended for international partnerships; if all your authors are based in the area of study, this question is not applicable.

   This should include a thorough description of their leadership role(s) in the study. Are local researchers named in the author list or the acknowledgements, or are they not mentioned at all (and, if not, why)? Please also describe the involvement of early career researchers based in the location of the study. Some of this information might be repeated from the Contributors section in the manuscript. Note: we adhere to [ICMJE authorship criteria] when deciding who should be named on a paper.

**a) Study design:**<br>The study authors include researchers from the following teams:<br>- Nigeria: ALF, RY, FAb, LCA, HMS, AAW, HG<br>- Kenya: JKM, PO, JO, ZQ, AO, GG<br>- Tanzania: MS, AB, AM, FAA<br>- South Africa: FS, SW, SDK, MSM, EA, EM, SF, NM, GJF<br>- Trial sponsor (University of Birmingham, UK): KMM, AD, AC, JM, CE<br>- Other collaborators (WHO): FAl, IG<br>KMM, IG, AC conceptualised and designed this study. KMM, ALF, RY, FAb, LCA, JKM, PO, MS, AB, FS, SW, and SDK were involved in data curation and KMM, ALF, RY, FAb, LCA, HMS, AAW, JO, AM, MSM, EA, EM, and AD involved in project administration. Those named in the Acknowledgements section contributed to data collection, but did not meet the ICMJE criteria for authorship.
**b) Clinical study processes:**<br>The study implementation was supervised by KMM, HG, ZQ, FAA, MSM, SF and NM and were responsible for overseeing study processes in each site. KMM conducted online training with in-country support from JO, AM, HMS, AAW, MSM, EA, and EM.<br>ALF, RY, FAb, LCA, JKM, PO, MS, AB, FS, SW, SDK, and those listed in the acknowledgements collected the data. ALF, RY, FAb, LCA, HMS, AAW, JO, AM, MSM, EA, EM provided in-country support for the logistics and data collection.
**c) Data interpretation:**<br>AD built the REDCap database. Data management was coordinated by KMM, with in-country support from ALF, JO, MSM, EA, EM, AM. KMM cleaned the dataset and conducted the initial data analysis and visualisations with support from JM. All authors contributed to the interpretation of the data presented in the manuscript.
**d) Manuscript preparation:**<br>KMM wrote the original draft. All authors contributed to interpretation of results and revised the manuscript. KMM and AC accept responsibility for the decision to submit for publication.

2. Were the data used in your study collected by authors named on the paper, or have they been extracted from a source such as a national survey? ie, is this a secondary analysis of data that were not collected by the authors of this paper. If the authors of this paper were not involved in data collection, how were data interpreted with sufficient contextual knowledge?

   The Lancet Global Health *believe contextual understanding is crucial for informed data analysis and interpretation.*

Data used in this study consisted of observations of vaginal birth and PPH. All data presented in this paper were collected for the purposes of this study, and is therefore primary data. Those named as co-authors are from the study context and provided critical insights into interpretation of the study context and results.

3. How was funding used to remunerate and enhance the skills of researchers and institutions based in the area(s) of study? And how was funding used to improve research infrastructure in the area of study?

   Potentially effective investments into long-term skills and opportunities within institutions could include training or mentorship in analytical techniques and manuscript writing, opportunities to lead all or specific aspects of the study, financial remuneration rather than requiring volunteers, and other professional development and educational opportunities.

   Improvements to research infrastructure could be funding of extended trial designs (such as platform trials) and use of master protocols to enable these designs, establishment of long-term contracts for research staff, building research facilities, and local control of funding allocation.

**Skills:**<br>The Kenya, Nigeria, South Africa and Tanzania teams worked closely on all phases of the E-MOTIVE study design and intervention implementation with the mixed-methods team, trial sponsor, and other collaborators, and strengthened research capacity related to protocol design, research ethics considerations (particularly for cluster randomised trials and observational studies), data management and analysis, academic writing, and preparation of study derivative products and implementation into policy (e.g. the WHO postpartum haemorrhage [PPH] guidelines).
**Research infrastructure:**<br>The E-MOTIVE project has contributed to strengthening research capacity in the context of clinical trials, mixed-methods evaluation of trials, and economic evaluation at the four study sites, including conducting and generating evidence on priority areas of maternal health conditions and implementation of effective clinical practices. Following the demonstration of effectiveness of the E-MOTIVE intervention, all control sites moved to an implementation phase, to ensure that all study sites have access to life-saving interventions and training.

4. How did you safeguard the researchers who implemented the study?

   Please describe how you guaranteed safe working conditions for study staff, including provision of appropriate personal protective equipment, protection from violence, and prevention of overworking.

All institutions involved in the E-MOTIVE project (Bayero University, University of Cape Town, KwaZulu-Natal Department of Health, University of the Witwatersrand, University of Nairobi,

Muhimbili University of Health and Allied Sciences, University of Birmingham, and WHO) have policies on safe working conditions, equal employment, human resources, and protection from violence, overworking and exploitation. Data collectors within the health facilities and study staff during monitoring visits were provided with appropriate transportation and personal protection (e.g. face masks, hand sanitizer during COVID-19).

Benefits to the communities and regions of study

5. How does the study address the research and policy priorities of its location?

How were the local priorities determined and then used to inform the research question? Who decided which priorities to take forward? Which elements of the study address those priorities?

How does the study address the research and policy priorities of its location?
Kenya, Nigeria, South Africa, and Tanzania have high maternal mortality rates due to PPH, making maternal health a key focus in their national health strategies. The E-MOTIVE study aligns with these priorities by evaluating strategies to improve early detection and management of PPH, thereby addressing a critical gap in maternal healthcare.
How were the local priorities determined and then used to inform the research question?
Local priorities were identified through national health policies, maternal mortality data, and engagement with key stakeholders, including policymakers, healthcare providers, and researchers. Existing gaps in clinical practice, such as inconsistent use of blood loss measurement tools and delayed PPH detection, were highlighted during these discussions. These insights informed the research question, focusing on whether a standardised, early detection and treatment approach could improve maternal outcomes.
Who decided which priorities to take forward?
The selection of priorities was guided by consultations with maternal health experts, and clinical practitioners in each country. Additionally, alignment with global maternal health recommendations, including WHO guidelines on PPH management, ensured that the study addressed both local and international priorities.
Which elements of the study address those priorities?
The study addresses these priorities by providing evidence to support policy and practice changes. Integrating findings into clinical guidelines, obstetric and midwifery education, and ongoing training can enhance early PPH detection and response. Additionally, adopting calibrated blood loss measurement tools and globally aligned diagnostic criteria, while allowing for context-specific adaptations, can improve diagnostic accuracy. These strategies are important in low- and middle-income countries, where strengthening early detection and intervention can significantly enhance maternal health outcomes.

6. How will research products be shared in the community of study?

For instance, will you be providing written or oral layperson summaries for non-academic information sharing? Will study data be made available to institutions in the region(s) of study? The Lancet Global Health *encourages authors to translate the summary (abstract) into relevant languages after paper editing; do you intend to translate your summary?*

Findings from the E-MOTIVE study have been disseminated at a national level to all key stakeholders in Kenya, Nigeria, South Africa and Tanzania, and with all 78 trial intervention and control sites. The control sites have implemented the E-MOTIVE intervention to ensure that the calibrated drape and MOTIVE bundle are implemented in practice, with ongoing efforts expanding

training to all hospitals as standard practice. Lay person summaries in local languages have been prepared for the E-MOTIVE programme of work. The positive findings from the E-MOTIVE study have already been translated into updated WHO guideline recommendations (2023), which are now being adapted and implemented at the country level in the four study countries and beyond.

7. How were individuals, communities, and environments protected from harm?

a) *How did you ensure that sensitive patient data was handled safely and respectfully? Was there any potential for stigma or discrimination against participants arising from any of the procedures or outcomes of the study?*

The identities of all study participants are anonymous and no identifiable information was collected.

b) *Might any of the tests be experienced as invasive or culturally insensitive?*

Not applicable.

c) *How did you determine that work was sensitive to traditions, restrictions, and considerations of all cultural and religious groups in the study population?*

Not applicable – the E-MOTIVE intervention components are all considered as standard practices of care.

d) *Were biowaste and radioactive waste disposed of in accordance with local laws?*

Yes, the obstetric drape containing blood was disposed of following local policies and procedures according to local laws.

e) *Were any structures built that would have impacted members of the community or the environment (such as handwashing facilities in a public space)? If so, how did you ensure that you had appropriate community buy-in?*

Not applicable.

f) *How might the study have impacted existing health-care resources (such as staff workloads, use of equipment that is typically employed elsewhere, or reallocation of public funds)?*

Delivering the E-MOTIVE intervention may have increased staff workloads – this was included as part of the E-MOTIVE process evaluation and found that >69% of survey participants did not think that it increased their workload. The E-MOTIVE process evaluation results show that health workers in general found the intervention highly acceptable, improved care for women, and reduced work-related stress.

8. Finally, please provide the title (eg, Dr/Prof, Mr/Mrs/Ms/Mx), name, and email address of an author who can be contacted about this statement. This can be the corresponding author.

Name: Ms Kristie-Marie Mammoliti
Email: kmammoliti@unimelb.edu.au
